# Targeting protein myristoylation for the treatment of prostate cancer

**DOI:** 10.18632/oncoscience.391

**Published:** 2018-01-22

**Authors:** Essilvo Sulejmani, Houjian Cai

**Affiliations:** Department of Pharmaceutical and Biomedical Sciences, College of Pharmacy, University of Georgia, Athens,Athens, GA 30602, USA

**Keywords:** N-myristoyltransferase, myristoyl-CoA, B13, prostate cancer, Src kinase

Chemotherapeutic agents for the treatment of various stages of prostate cancer have shown promising effects on overall survival of the patient. Emerging in 2004, the taxanes docetaxel and cabazitaxel have become the standard chemotherapeutic approaches. These agents have been explored for treatment of metastatic castration-sensitive or resistant prostate cancer. Docetaxel in combination with androgen deprivation therapy has consistently shown improvement in the overall survival for eligible patients with high-volume or earlier stages of metastatic castration-sensitive prostate cancer. Cabazitaxel has been used mainly for post-docetaxel treatment or following resistance to docetaxel [1]. While the future of chemotherapy treatments will focus on the combination of drugs to mitigate cross-resistance, more options for treatments are urgently needed to improve patient survival rate.

N-myristoyltransferase (NMT) catalyzes a co/post-translational modification that leads to the covalent attachment of the myristoyl-group (14-carbon saturated fatty acyl) to the N-terminus of a target protein. A consensus sequence of Met-Gly2-x-x-x-Ser/Thr_6_ in the N-terminus is found in target proteins, with the required N-terminal Gly residue serving as the site of attachment of the myristoyl-group after the first methionine is removed by methionine aminopeptidase during protein translation. Two mammalian NMT isoforms, NMT1 and NMT2, share 77% protein identity. The enzymatic kinetics of NMT1 has an ordered Ping-Pong Bi-Bi mechanism, which requires the binding of myristoyl-CoA before the binding of the protein substrate [2,3]. The N-terminal half of NMT enzymes forms the majority of the myristoyl-CoA binding site, providing several important contacts for the protein substrate binding site as well; the C-terminal half contributes to the majority of the protein substrate binding site, and only aids in the binding of myristoyl-CoA to the N-terminal half by the side chain of Phe425. Myristoyl-CoA bound to NMT1 contains several bends: at the C6-C7 pantetheine, and at C1 and C5-C6 of myristate. The Phe170 and Leu171 residues of NMT1 form an oxyanion hole to which the C1-O1 carbonyl of the myristoyl-CoA is attracted (Fig.1) [2]. Protein myristoylation usually occurs co-translationally during *de novo* protein synthesis. Recently, numerous NMT target proteins have also been identified when a consensus sequence at the N-terminus was exposed after being cleaved by a protease [4]. While many studies have demonstrated that myristoylation of the proteins such as Src family kinases, AMPK, and others is essential for their molecular functions, further studies should evaluate biological functions of the myristoylation on the protease-processed products.

**Figure 1:**
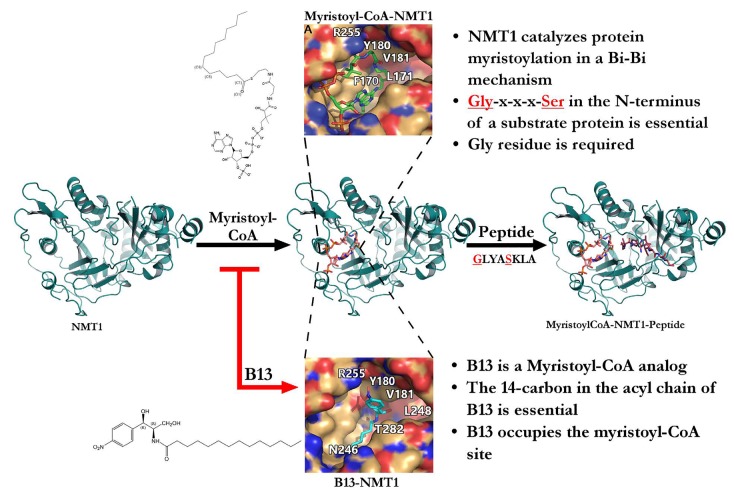
B13 binds to the myristoyl-CoA binding site to inhibit NMT1 enzymatic activity and prostate cancer progression

Both the myristoyl-CoA and the protein-binding site of NMTs could serve as targets to inhibit the myristoylation process. Kim et al. have demonstrated an effective approach to inhibit NMT1 activity by blocking the myristoyl-CoA binding site, and serve as an antitumor target for inhibiting proliferation of prostate cancer cells. B13 and LCL204 are structural analogs of myristoyl-CoA that inhibit NMT1 enzymatic function: B13 competes with myristoyl-CoA for the NMT1 myristoyl-CoA binding site due to its similar hydrophobic, 14-carbon chain tail [3]. Molecular docking analysis indicates that several favorable interactions occur between the B13 and the NMT1 binding pocket. The interactions include: 1) the aromatic ring of B13 with a hydrophobic patch comprised of NMT1's Tyr180 and Val181; 2) B13's aliphatic tail with NMT1's Asn246; 3) hydrogen bonding between B13's R2 nitro group and NMT1's Arg255, B13's amide group and NMT1's Thr282 and the backbone amide of Leu248, and B13's R4 hydroxymethyl group and the hydroxyl group of NMT1's Tyr180 (Fig.1). Targeting NMTs will potentially inhibit the activity of a variety of myristoylated proteins, including some of the oncogenic driver gene products. For example, studies focusing on the catalysis of myristoylation have proven to be valuable resources in the demonstration of Src kinase activity [3]. Elevated levels of Src kinase are found in advanced stages of prostate cancer such that higher Src kinase levels correlate with prostate cancer cell proliferation and tumor aggression. B13 has proven to be a successful NMT1 inhibitor for Src-driven prostate cancer by preventing Src localizing at the membrane and subsequent Src kinase mediated oncogenic signaling. Therefore, this compound inhibits the NMT1-Src axis, which mediates the proliferation of prostate cancer cells and Src-driven tumor progression [3].

NMT may serve as a specific target for inhibiting the progression of prostate tumors, as genetic and pharmacological inhibition of NMT suppresses the proliferation of prostate cancer cells but not normal cells [3]. The differential inhibitory effect might rely on the unique aberrant elevation of fatty acid metabolism in prostate cancer cells. An array of enzymes is reprogrammed to up-regulate *de novo* fatty acid biosynthesis including: ATP citrate lyase, acetyl-CoA carboxylase α, and fatty acid synthase in the fatty acid biosynthesis pathway [5,6]. Prostate cancer cells also have complementary pathways to break down stored triacylglycerols and cholesterol esters, such as the elevated activity of monoacylglycerol lipase. The aberrant fatty acid synthesis and lipolytic pathways provide a variety of fatty acid metabolites to enhance oncogenic signaling in prostate cancer cells [5]. As one of the major acyl-CoA metabolites [7], myristoyl-CoA potentially increases the myristoylation of proteins [8]. For example, experimental evidence suggests that exogenous MA leading to noticeable elevation of myristoyl-CoA levels is a rate limiting factor for increasing myristoylated Src, which significantly promotes the Src-mediated MAPK and FAK oncogenic signaling *in vitro* and correlated with acceleration of Src mediated prostate tumor progression in a high fat diet [8]. Therefore, targeting NMT enzymatic function represents a potential chemotherapeutic option for prostate cancer, and exhibits specificity on suppressing prostate cancer cells.

## References

[1] Bhatnagar RS (1999). Biochim Biophys Acta.

[2] Kim S (2017). Cancer Res.

[3] Kim S (2017). J Biol Chem.

[4] Quinn DI (2017). Ann Oncol.

[5] Rohrig F, Schulze A (2016). Nat Rev Cancer.

[6] Thinon E (2014). Nat Commun.

[7] Yang X (2017). Anal Chem.

[8] Zadra G (2013). Biochim Biophys Acta.

